# Localized renal artery vasculopathy: avoiding misdiagnosis between inflammatory arteritis and isolated renal artery dissection

**DOI:** 10.1007/s10067-026-08246-0

**Published:** 2026-07-13

**Authors:** Sopie Secka, Ilaria Calciolari, Beatrice Maranini, Alfonso Massara, Andrea Lo Monaco, Filippo Furicchia, Zairo Ferrante, Pierfilippo Acciarri, Alessio Mario Cosacco, Aaron Thomas Fargion, Marcello Govoni

**Affiliations:** 1 Rheumatology Unit, Azienda Ospedaliero-Universitaria Sant’Anna, University of Ferrara, Ferrara, Italy; 2 Department of Interventional and Diagnostic Radiology, Arcispedale Sant’Anna, Ferrara, Italy; 3 Unit of Vascular and Endovascular Surgery, Thoraco-Cardio-Vascular Department, University of Ferrara, Ferrara, Italy

**Keywords:** Angiography, Inflammatory arteritis, IRAD, Renal artery vasculopathy, Vasculitis

## Abstract

**Background:**

Isolated renal artery involvement, whether inflammatory or dissecting, is an uncommon and challenging presentation in clinical practice. Its nonspecific symptoms and overlap with non-inflammatory vasculopathies complicate the distinction between localized vasculitis and isolated renal artery dissection (IRAD), two entities requiring fundamentally different therapeutic approaches.

**Case presentation:**

We report two cases of unilateral renal artery pathology initially suspected to represent vasculitis. Case 1: A 47-year-old man presented with acute flank pain. CT and MRA revealed perivascular tissue surrounding the left renal artery, a small pseudoaneurysm, and patchy hypoperfusion. Serology, inflammatory markers, and PET/CT excluded systemic vasculitis. Corticosteroid therapy led to complete radiologic resolution, consistent with a localized idiopathic fibro-inflammatory arteritis. Case 2: A 42-year-old patient developed abrupt-onset hypertension and renal ischemia. Initial imaging suggested “sleeve-like” arterial thickening, raising concern for vasculitis. Selective angiography subsequently confirmed spontaneous renal artery dissection with thrombotic involvement of a segmental branch. The patient was managed conservatively with antihypertensives and antiplatelet therapy, achieving clinical stability and partial perfusion recovery.

**Discussion:**

These cases illustrate the diagnostic complexity of isolated renal artery disease. Normal or only mildly elevated inflammatory markers do not reliably exclude localized vascular inflammation. Advanced cross-sectional imaging and timely angiography are essential when diagnostic uncertainty persists. Misinterpretation may result in unnecessary immunosuppression or failure to recognize IRAD.

**Conclusion:**

A structured, multidisciplinary evaluation integrating serologic assessment, high-resolution imaging, and close follow-up is crucial for accurate diagnosis. While isolated vasculitis may require immunosuppression, IRAD typically responds to conservative therapy, underscoring the need for individualized management.

## Introduction

Vasculitis encompasses a heterogeneous group of disorders characterized by inflammation of blood vessel walls, leading to structural damage such as luminal narrowing, aneurysm formation, thrombosis, and downstream organ ischemia [1]. Classification schemes generally divide vasculitis based on the size of affected vessels—large, medium, or small—and their clinical, histopathological, and immunological features [2].

Renal involvement is common in medium- and small-vessel vasculitis, typically manifesting as necrotizing glomerulonephritis in the context of systemic disease. However, vasculitis involving renal arteries without glomerular involvement or systemic symptoms is exceedingly rare and remains poorly understood. Such cases, often presenting with isolated renal infarction, renovascular hypertension, or incidental imaging findings, pose significant diagnostic challenges due to their nonspecific clinical features and overlap with other vascular conditions [3].

Among vascular mimickers, isolated renal artery dissection (IRAD) is an important differential diagnosis. IRAD is a rare vascular emergency with an often spontaneous etiology, most frequently affecting middle-aged men. It can present with acute flank pain and hypertension, mimicking renal colic, infarction, or even vasculitis. In both IRAD and isolated vasculitis, imaging—especially computed tomography angiography (CTA) or magnetic resonance angiography (MRA)—plays a pivotal role in diagnosis, while the absence of systemic inflammatory markers may hinder early recognition [4].

The literature remains limited regarding the presentation, management, and outcomes of patients with isolated renal artery involvement due to vasculitis or dissection. The differentiation between inflammatory and non-inflammatory arterial lesions is crucial, as it directly influences therapeutic decisions—ranging from immunosuppression to conservative medical management or vascular intervention.

In this report, we present two cases of patients with unilateral renal artery pathology, initially suspected to have vasculitis based on imaging, who were ultimately managed with different strategies following multidisciplinary assessment. Through these cases, we aim to highlight the diagnostic complexity and management considerations of rare renal artery disease, with reference to published literature.

## Case report no. 1

In October 2023, a 47-year-old man presented to the Emergency Department (ED) with acute onset of left flank pain. His past medical history included colonic diverticulosis, chronic rhinopathy due to left-sided nasal septal deviation, and bronchial asthma since childhood.

Initial evaluation in the ED revealed normal serum creatinine and C-reactive protein (CRP) levels. An abdominal X-ray showed fecal impaction without signs of free air. He was subsequently discharged.

**Fig. 1:**
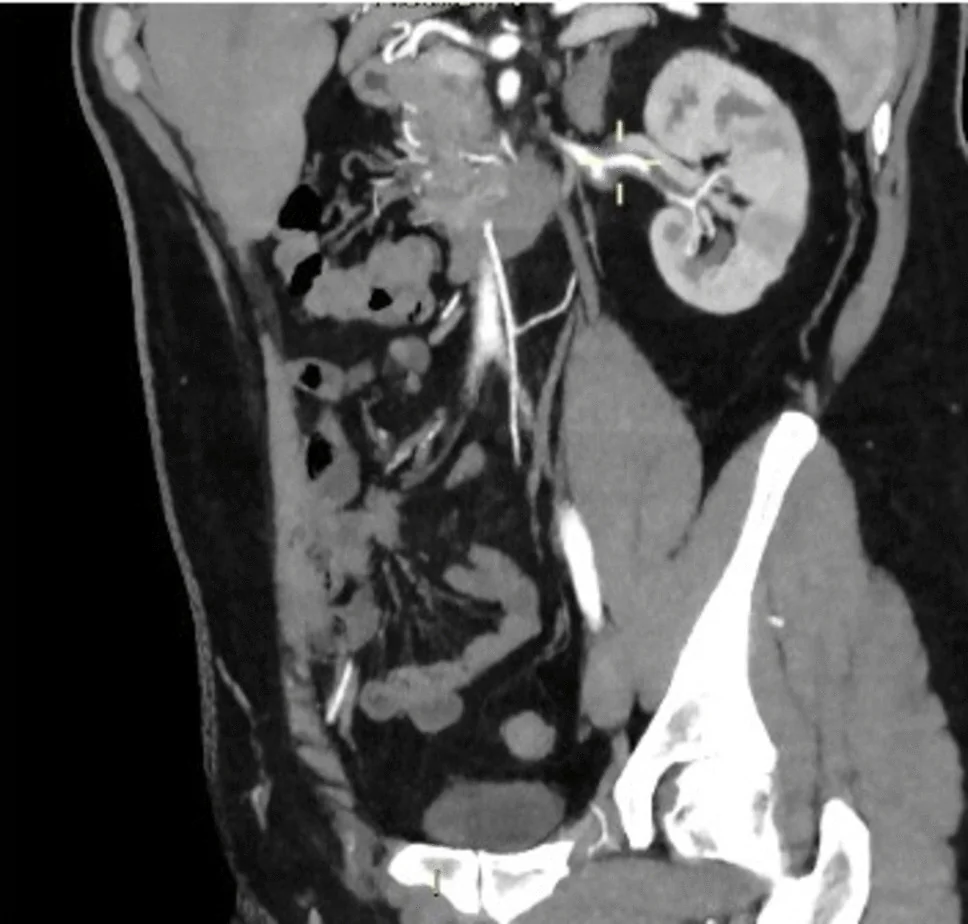
Hyperdense perivascular tissue surrounding the left renal artery near its bifurcation, with no evident contrast enhancement

Following a gastroenterological consultation for suspected diverticulitis, a contrast-enhanced abdominal CT scan was performed. The scan did not confirm diverticulitis but incidentally revealed a hyperdense perivascular tissue surrounding the left renal artery near its bifurcation, with no evident contrast enhancement (Fig. 1). The vascular branches appeared slightly reduced in caliber, and the perivascular abnormality extended into the intrarenal region. A 5-mm saccular arterial-phase enhancement, initially suggestive of a pseudoaneurysm, was also noted. Additionally, multiple areas of hypoperfusion were identified in the left kidney, which was otherwise normal in size and showed no evidence of calculi. Mild splenomegaly (12.6 cm) and sigmoid diverticulosis were also reported.

**Fig. 2:**
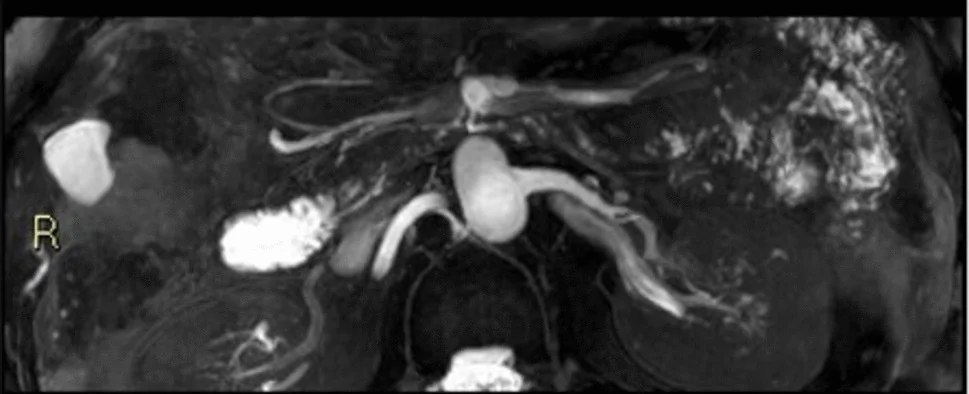
Perivascular cuffing of the left renal artery, extending from its origin to the hilum along the main lobar and interlobar branches, with mild hypointensity on T2-weighted images, and no significant diffusion restriction on DWI sequences

Further evaluation with MRA demonstrated perivascular cuffing of the left renal artery, extending from its origin to the hilum along the main lobar and interlobar branches (Fig. 2). This tissue showed mild hyperintensity on T1-weighted images, hypointensity on T2-weighted images, and no significant diffusion restriction on DWI sequences. The pseudoaneurysm at the arterial bifurcation was confirmed, along with cortical T2-hypointense areas in the lower pole of the left kidney, consistent with hypoperfusion.

The patient was subsequently admitted to the Rheumatology Department for further investigation in the suspicious of localized vasculitis.

During hospitalization, he remained asymptomatic, reporting no abdominal pain or systemic symptoms.

Laboratory evaluation revealed normal blood count, renal and hepatic function, and no signs of systemic inflammation. Serologic testing was negative for hepatitis B and C. Antinuclear antibodies (ANA) were positive with a cytoplasmic (anti-cytoskeleton) pattern, considered nonspecific. Anti-neutrophil cytoplasmic antibodies (ANCA), extractable nuclear antigen (ENA) panel, and complement levels were all within normal ranges. Complement levels (C3, C4), Vidal-Wright serodiagnosis, and Quantiferon test were negative. IgG subclass levels (IgG1, IgG2, IgG3, IgG4) were within normal limits. HLA class I typing was not informative. Anti-cardiolipin antibodies, anti-beta-2-glycoprotein I antibodies, and lupus anticoagulant (LAC) were negative.

The case was discussed with the Radiology Consultant, who initially considered the findings to be suggestive of a vasculitic process. The hypothesis of renal artery dissection was considered less likely, given the involvement of both arterial branches and the extension into the intraparenchymal vessels. To rule out large-vessel involvement, a PET/CT scan was performed, which did not reveal any areas of significant uptake.

Based on the available findings, the picture remained of uncertain interpretation but was considered most consistent with an idiopathic fibro-inflammatory process, localized exclusively to the main branches of the left renal artery.

In order to prevent potential complications, treatment with prednisone at a dose of 0.6 mg/kg was initiated (prednisone 50 mg daily).

**Fig. 3:**
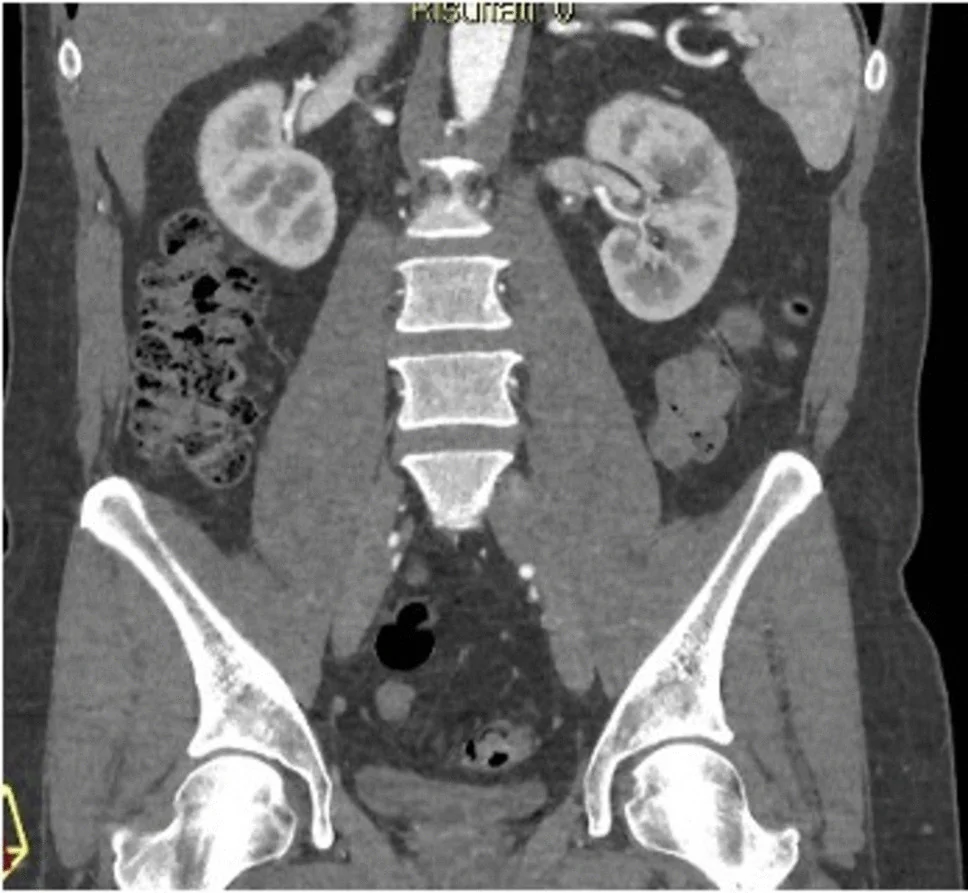
One-month CT scan: no longer detectable hypodense thickening of left renal artery

A follow-up contrast-enhanced CT scan performed approximately one month later, under prednisone 43.75 mg daily, showed that the previously noted hypodense thickening of the left renal artery and its branches were no longer detectable (Fig. 3). The patient then rapidly tapered steroid (leaving 6.25 mg every week reaching 5 mg daily at 6 months from baseline CT scan).

At 1 year follow-up CT, the previously reported perivascular hypodense tissue surrounding the left renal artery was still no longer detectable, nor the previously reported areas of kidney hypoperfusion. Notably, a marked reduction in the size of the pseudoaneurysm at the bifurcation of the left renal artery was described, which is now millimetric. Steroid was then discontinued.

## Case report no. 2

A previously healthy 42-year-old patient presented in late March 2025 with episodic bilateral temporo-parietal headaches accompanied by paraesthesia, xerostomia, gastroesophageal reflux disease (GERD)–like symptoms, low-grade fever, and generalized fatigue. On April 2nd, the patient was evaluated in ED, where intravenous paracetamol (1 g) was administered with resolution of the headache.

The following day, the patient returned with new-onset left flank pain and reported elevated blood pressure at home (> 170/100 mmHg) over the prior 2–3 days. There were no associated respiratory, visual, or abdominal symptoms, and the patient denied jaw claudication, limb claudication, or abdominal angina.

**Fig. 4:**
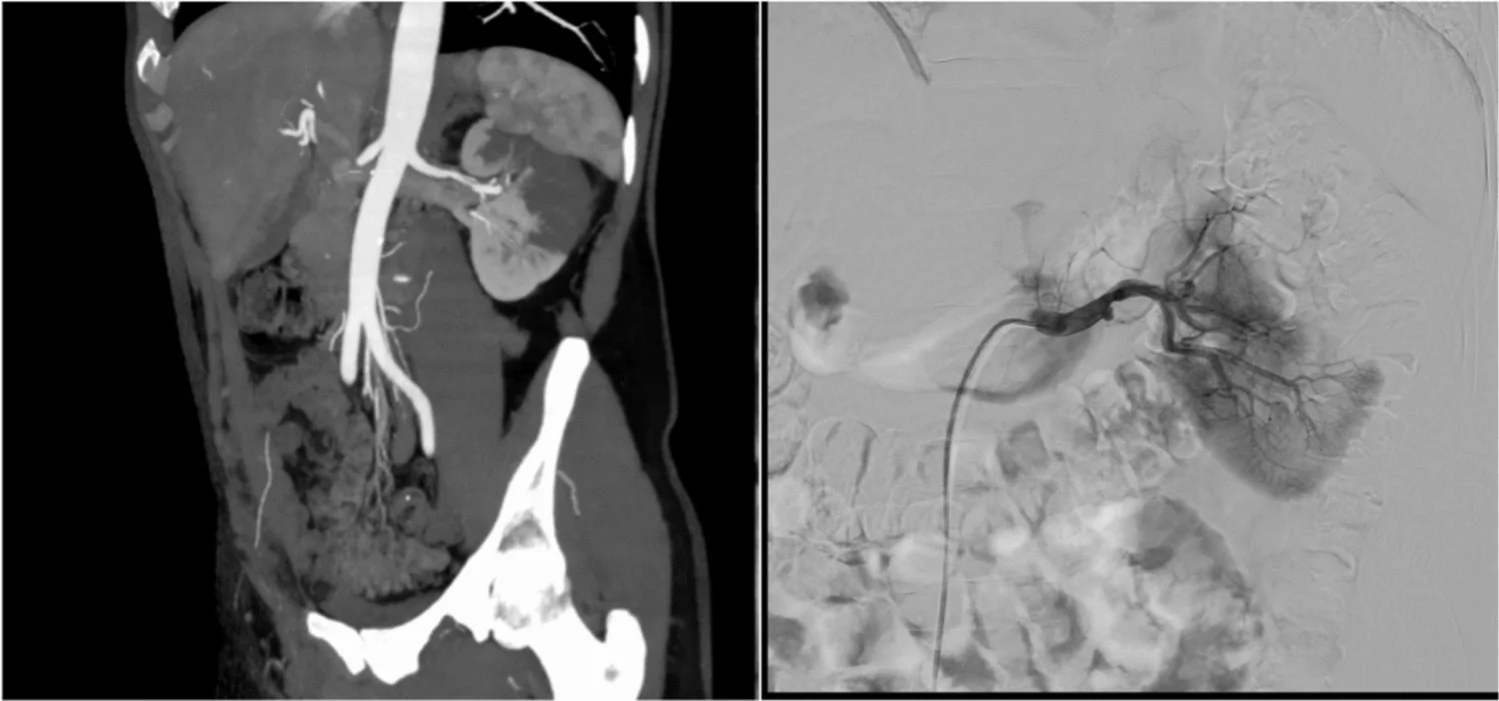
On the left: contrast enhancement defect in the mid-to-upper third of the left kidney, with an infarct-like appearance in the mid-third on CT scan. On the right: angiography showing left renal artery of reduced caliber with trombotic deposition

A contrast-enhanced abdominal CT scan (Fig. 4) revealed a hypodense area in the mid-third of the left kidney consistent with renal ischemia, along with a filiform appearance of the left renal artery. Rheumatology consultation raised concern for possible renal artery vasculitis, prompting hospital admission.

During hospitalization, the patient remained hemodynamically stable. Physical examination revealed normal radial pulses but reduced right dorsalis pedis and left posterior tibial pulses. The abdomen was soft and non-tender.

Laboratory tests showed elevated inflammatory markers, with a CRP of 3.7 mg/dL. In the context of CT findings suggestive of “sleeve-like thickening” of the left renal artery, large-vessel vasculitis was suspected. High-dose IV methylprednisolone (250 mg daily for 3 days) was administered, followed by oral prednisone 75 mg/day.

Hypertension was the predominant clinical finding. Antihypertensive therapy with amlodipine 5 mg twice daily, doxazosin 2 mg twice daily, and bisoprolol 1.25 mg daily was initiated, leading to progressive improvement in blood pressure control.

A whole-body PET scan revealed no evidence of metabolically active vasculitis. Doppler ultrasound of the upper and lower limb arteries and supra-aortic trunks showed no significant stenosis or occlusions.

Given ongoing diagnostic uncertainty and the abrupt onset of symptoms, selective angiography of the left renal artery was performed. It demonstrated a narrowed segment distal to the main bifurcation, with thrombotic material in the inferior branch, findings suggestive of a renal artery dissection (Fig. 4). Compared to the previous CT, the degree of narrowing was less pronounced. The hilar arterial ectasia remained unchanged, and the renal ischemic findings were substantially stable, with persistent ischemia involving the mid-third and upper pole of the left renal parenchyma.

The case was subsequently re-evaluated in a multidisciplinary discussion with radiology and vascular surgery teams, confirming the diagnosis of spontaneous renal artery dissection. Given the complete thrombosis of the false lumen and the presence of renal ischemic changes, an endovascular revascularization approach was excluded. Medical management was continued, including blood pressure control and low-dose aspirin for antithrombotic therapy. The clinical course was favorable, with no recurrence of abdominal pain, progressive improvement in blood pressure control, and preservation of normal renal function.

A follow-up contrast-enhanced abdominal CT scan was scheduled at 2 weeks to monitor the post-stenotic dilation downstream of the dissection site.

A gradual tapering of corticosteroid therapy was initiated (the patient was discharged under prednisone 25 mg daily, withdrawing 5 mg every 5 days until suspension in 20 days).

**Fig. 5:**
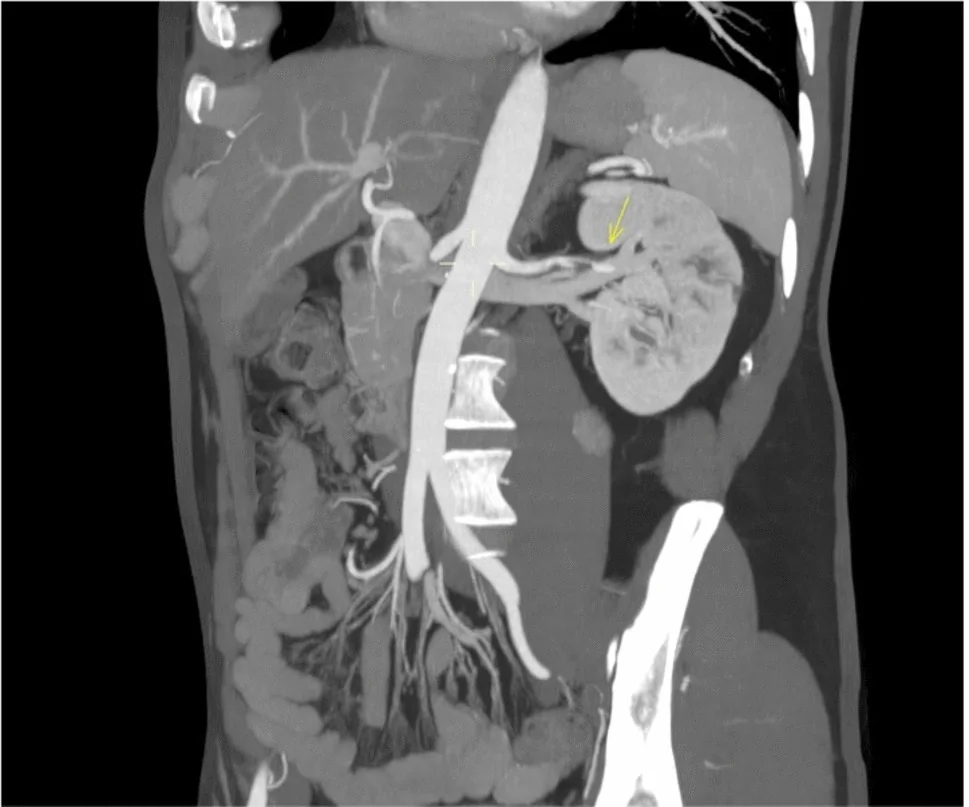
Thrombotic stenosis at the level of the left renal artery in its lower portion, stable, and ectasia at the ilar site, slightly reduced. Improvement in the perfusion of the related subsegmental branches, with a knowm ischemic area. Arrow: residual post-stenotic ectasia

At 2-week follow-up, a contrast-enhanced PET/CT confirmed stability of the left renal artery dissection, located just distal to the main bifurcation, with persistent thrombotic stenosis of the inferior branch and slight reduction in hilar ectasia (Fig. 5). Importantly, there was improved perfusion of affected subsegmental branches and partial recovery of left renal parenchymal perfusion, albeit still delayed. A residual ischemic area in the mid and upper pole of the left kidney was stable in extent and less hypodense, mainly visible in the venous phase. No hydronephrosis was observed. The contralateral kidney and other abdominal organs appeared normal. There were no signs of active vasculitis or malignancy. These findings supported continuation of conservative management.

## Discussion

Isolated renal artery involvement, whether due to vasculitis or dissection, is a rare and diagnostically challenging condition, given its nonspecific presentation [5]. Only a limited number of similar cases have been described in the literature, reflecting the rarity and under-recognition of isolated renal artery vasculitis and spontaneous renal artery dissection as distinct clinical entities [6]. The two presented cases underscore this diagnostic dilemma, each initially suspected to represent vasculitic involvement, yet diverging toward different etiologic conclusions and management paths.

The differential diagnosis between isolated renal vasculitis and spontaneous isolated renal artery dissection (IRAD) is clinically critical, as the two conditions, while potentially presenting with similar symptoms (e.g., flank pain, renovascular hypertension), demand fundamentally different management strategies. The absence of systemic inflammatory markers or multisystem involvement should raise suspicion for non-vasculitic causes such as IRAD, fibromuscular dysplasia (FMD), or segmental arterial mediolysis [7]. A normal or only mildly elevated CRP level is often interpreted as evidence against active vasculitis. However, this assumption may not hold in cases of isolated or localized vascular involvement. On a speculative basis, vascular inflammation confined to a single organ, without glomerular involvement or constitutional symptoms, may not stimulate hepatic production of CRP in the same way as systemic vasculitis. Thus, low CRP values should not be used in isolation to rule out vasculitis, especially when imaging findings are suggestive of perivascular inflammation or pseudoaneurysm formation. Follow-up imaging and close monitoring remain essential in such uncertain scenarios to refine diagnosis and guide therapy, as in the first described case, which exhibited radiological features suggestive of perivascular inflammation of the left renal artery and its intrarenal branches, raising suspicion for isolated renal vasculitis. However, PET/CT excluded active vasculitis or other large-vessel involvement.

PET imaging, particularly FDG-PET/CT, has emerged as a valuable adjunct in the evaluation of suspected isolated renal artery vasculitis, especially when standard sierologic and clinical markers are inconclusive. In the first case, PET/CT played a pivotal role by demonstrating the absence of fluorodeoxyglucose (FDG) uptake in the renal artery wall and other large vessels, effectively ruling out active vasculitis or systemic involvement. This helped refine the diagnosis and prevent unnecessary escalation of immunosuppression. PET’s utility lies in its ability to detect metabolically active inflammation even when structural changes are subtle or absent on conventional imaging, thereby offering functional insight beyond the anatomic findings of CTA or MRA. However, its sensitivity may be limited in cases of small-vessel involvement or low-grade, localized inflammation, highlighting the need to interpret PET findings within the broader clinical and imaging context. Despite these limitations, when used in conjunction with cross-sectional imaging and serology, PET/CT can enhance diagnostic confidence and assist in guiding duration and intensity of therapy in complex cases of renal artery pathology [8–10].

These findings parallel those reported in a rare form of isolated, unilateral renal vasculitis, characterized by localized inflammation without systemic involvement or glomerular disease [5, 11, 12]. In such cases, imaging, particularly contrast-enhanced MRI or CT, may show perivascular tissue with hypoperfusion, mimicking other causes of renovascular compromise.

Histologic confirmation is generally considered the gold standard in vasculitis, but renal artery biopsy carries substantial risk and was not pursued here [13]. Instead, the patient’s complete radiological and clinical response to corticosteroids, with regression of perivascular tissue and improvement in pseudoaneurysm, supports a self-limited, likely immune-mediated inflammatory process. While this may not fulfil formal criteria for systemic vasculitis, the presentation is consistent with localized, idiopathic arteritis, a diagnostic gray zone with overlapping features with fibrosing vasculopathies.

The second case revealed radiologic findings of a narrowed left renal artery with post-stenotic ectasia and thrombus in a segmental branch, highly suggestive of renal artery dissection. Although initially suspected to be vasculitic in nature, selective angiography clarified the diagnosis, and the patient’s course was managed conservatively with blood pressure control, low-dose aspirin, and steroid taper until rapid suspension, avoiding unnecessary long-term steroid course, and in line with first strategy approach suggested by the latest 2025 European Vascular Surgery Society guidelines [14, 15].

This aligns with findings from the systematic review by Jha et al. [4], which documented 129 cases of isolated renal artery dissection. Most cases occurred in middle-aged men, frequently with hypertension but without systemic inflammatory disease. In the reviewed cohort, spontaneous etiology accounted for 76% of IRAD cases, with abdominal pain and renovascular hypertension as common presentations, both observed in our patient. Imaging, particularly CTA and MRA, remains the cornerstone for diagnosis, with angiography reserved for uncertain or evolving scenarios [4].

In terms of management, conservative treatment, including antihypertensives and antithrombotic, was the most common strategy and yielded favorable outcomes in over 90% of cases [4]. This patient similarly demonstrated stable imaging findings and partial perfusion recovery with conservative therapy.

Both cases underscore the importance of maintaining a broad differential diagnosis in patients presenting with flank pain and renal hypoperfusion. While glomerular vasculitis dominates renal involvement in systemic vasculitides, isolated arterial involvement is rare and can be misdiagnosed as dissection, fibromuscular dysplasia, or idiopathic inflammatory conditions [4, 5, 7].

Moreover, it is important to emphasize that the benefits of conservative therapy include a reduced risk of procedural complications, the ability to manage the underlying systemic disease, and good efficacy in blood pressure control and prevention of renal damage progression in most patients. Revascularization, on the other hand, may be considered only in situations of critical ischemia, acute deterioration of renal function, or uncontrolled hypertension despite optimal medical therapy, and should preferably be planned during the remission phase of vasculitis to minimize the risk of complications [16, 17].

Reported complications following revascularization of renal artery stenosis in patients with vasculitic or inflammatory processes include restenosis (up to 44% at 5 years in patients with Takayasu arteritis, more frequent after endovascular than surgical procedures), graft or stent thrombosis, bleeding (both local and systemic), worsening renal function or acute kidney injury—often related to embolization, contrast-induced nephropathy, or arterial occlusion—embolization (atheromatous or thrombotic), which may lead to renal infarction or peripheral organ damage, dissection or rupture of the renal artery, local vascular complications such as groin hematoma, pseudoaneurysm, arteriovenous fistula, or retroperitoneal haemorrhage, the need for surgical reintervention for organ salvage or bleeding, and perioperative mortality (estimated at approximately 1%) [18–22].

The Society of Interventional Radiology reports that major complications occur in 12–14% of cases, with higher rates observed in the presence of active inflammation at the time of the procedure [18, 19].

Randomized controlled trials and meta-analyses have not demonstrated a significant benefit of revascularization over medical therapy in terms of renal or cardiovascular events, or survival, except in selected cases with rapidly declining renal function or refractory hypertension [17, 21, 23].

While IRAD is increasingly recognized due to improved imaging modalities such as CTA and MRA, isolated renal vasculitis remains a diagnosis of exclusion, often inferred based on radiologic perivascular inflammation, pseudoaneurysms, and steroid responsiveness, as in Case 1. However, steroid responsiveness alone does not definitively exclude IRAD (as see in Case 2), as inflammation can occasionally accompany dissection-related remodelling.

A major challenge lies in the differentiation between inflammatory vasculopathy and non-inflammatory vascular diseases like IRAD or segmental arterial mediolysis [6, 24]. While invasive confirmation through biopsy is often avoided due to procedural risk, integration of advanced imaging and serial follow-up is critical in guiding both diagnosis and therapeutic decisions.

A comparison of our cases with existing literature is summarized in Table 1, illustrating key demographic, clinical, and imaging features, as well as outcomes, in relation to previously reported cases of IRAD and isolated renal vasculitis.

**Table 1:** Summary and comparison of clinical features in presented cases and literature reports

Feature	Case 1 (suspected vasculitis)	Case 2 (IRAD)	Isolated renal vasculitis—literature **(**Toubi et al. [12]**)**	Isolated PAN (Pourafshar et al. [5])	IRAD—literature (Jha et al. [4])
Age/gender	47/male	42/male	67/female	75/male	129 cases: mean 43 years (± 13), 79% male
Presentation symptoms	Left flank pain, incidental CT finding	Headache, flank pain, hypertension	Variable; flank pain, hematuria, hypertension	Hypertension and mild renal function impairment	Abdominal/flank pain (76%), hypertension (54%)
Imaging findings	Perivascular tissue, pseudoaneurysm	Arterial narrowing, thrombus, ectasia	Asymmetric renal uptake, perivascular changes	At CT partially obstructed left renal artery	CTA/MRA sensitivity > 90%; segmental involvement
Laboratory findings	No systemic inflammation; ANA +	CRP mildly elevated, otherwise unremarkable	No systemic inflammation	CRP mildly elevated	No consistent pattern
Systemic involvement	None	None	None	None	None
Final diagnosis	Idiopathic isolated vasculitis	Spontaneous renal artery dissection	Localized, isolated unilateral arteritis	Isolated PAN	Spontaneous IRAD (76%); often misdiagnosed
Treatment	Prednisone (0.6 mg/kg)	Prednisone (short course), antihypertensives	Nephrectomy	Prednisone 0.5 mg/kg/day	57% medical (antihypertensives, anticoagulation)
Outcome	Complete resolution, steroid tapered off	Stable perfusion, improved BP control	Favorable without need of steroid	Improvement in his hypertension and renal function	91% favorable with conservative therapy

*IRAD* isolated renal artery dissection, *PAN* polyarteritis nodosa, *CT* computed tomography, *MRA* magnetic resonance angiography, *ANA* antinuclear antibodies, *CRP* C-reactive protein, *BP* blood pressure

As illustrated in Tables 2 and 3, accurate diagnosis of renal artery pathology requires a high index of suspicion and a tailored imaging strategy. While CTA and MRA remain cornerstones of non-invasive assessment, angiography and intravascular imaging may be indispensable in ambiguous cases. Clinical-radiologic dissociation, such as imaging suggestive of vasculitis with normal serologies and lack of systemic signs, should prompt consideration of IRAD or other vascular mimics.

**Table 2:** Diagnostic clues suggesting IRAD *vs* renal vasculitis

Feature	Suggests IRAD	Suggests vasculitis
Onset of flank pain	Sudden, sharp	Subacute, possibly dull or nonspecific
Blood pressure	Often markedly elevated	May be elevated but variable
Inflammatory markers (CRP, ESR)	Normal or mildly elevated	Frequently elevated
ANCA/autoantibodies	Negative	May be positive (though not always)
CTA/MRA findings	Dissection flap, luminal narrowing	Perivascular inflammation, pseudoaneurysms
PET-CT uptake	Absent	May show uptake if systemic inflammation
Response to steroids	Variable; not diagnostic	Often rapid clinical/radiologic improvement
Biopsy	Rarely performed, not typically needed	Gold standard (if safely feasible)

*IRAD* isolated renal artery dissection, *CRP* C-reactive protein, *ESR* erythrocyte sedimentation rate, *ANCA* antineutrophil cytoplasmic antibodies, *CT* computed tomography, *MRA* magnetic resonance angiography, *PET-CT* positron emission tomography-computed tomography

**Table 3:** Stepwise imaging strategy in suspected renal artery disease

Modality	Utility	Limitations
Doppler ultrasound	First-line, non-invasive	Low sensitivity for dissection
CTA	High sensitivity (91%) for IRAD	May miss subtle intramural hematomas
MRA	Excellent for vessel wall imaging	Contraindicated in some renal failure cases
PET-CT	Detects metabolic activity (vasculitis)	Limited spatial resolution in vessels
Conventional angiography	Gold standard for IRAD confirmation	Invasive; risk of contrast-induced injury
Intravascular ultrasound	Precise wall imaging, not routine	Requires specialized equipment and expertise

*CTA* computed tomography angiography, *IRAD* isolated renal artery dissection, *MRA* magnetic resonance angiography, *PET-CT* positron emission tomography-computed tomography

Misclassification of vasculitis in isolated renal artery disease is not uncommon and may lead to prolonged immunosuppressive therapy with significant side effects. As shown in Tables 4 and 5, a comprehensive evaluation that incorporates clinical presentation, serologic profile, imaging interpretation, and timely follow-up is crucial for accurate diagnosis. In particular, early radiologic reassessment allows for therapy de-escalation in cases of resolving IRAD or non-inflammatory vasculopathies. This stepwise, multidisciplinary approach reduces the risk of unnecessary treatment and associated complications.

**Table 4:** Common mimickers of renal vasculitis

Condition	Key features	How to differentiate from vasculitis
IRAD	Sudden pain, dissection flap, thrombosis, normal labs	Confirmed by CTA/angiography; no systemic inflammation
Fibromuscular dysplasia (FMD)	“String of beads” appearance, young females	No inflammation; typical angiographic pattern
Segmental arterial mediolysis	Aneurysm, haemorrhage, older patients, no systemic signs	Pathologic arterial wall changes; no inflammatory markers
Atherosclerosis	Gradual stenosis, older patients, systemic vascular disease	Diffuse vascular disease; plaque seen on imaging
Iatrogenic injury	Post-procedure, contrast use, catheterization	Clear procedural history; localized dissection or trauma
Infectious vasculitis	Fever, systemic symptoms, positive cultures	Serologies, cultures, PET-CT may help differentiate

*IRAD* isolated renal artery dissection, *CTA* computed tomography angiography, *PET-CT* positron emission tomography-computed tomography

**Table 5:** Key imaging and clinical red flags against vasculitis diagnosis

Feature	Suggests alternative diagnosis
Unilateral renal artery involvement	Consider IRAD, FMD, SAM
Lack of systemic symptoms	Less likely systemic vasculitis
Normal or mildly elevated inflammatory markers	Inconsistent with active vasculitis
Negative autoantibodies (ANCA, ANA, etc.)	Weigh against autoimmune etiology
Absence of PET uptake	No active inflammation
Stable/improved imaging without treatment	Consider non-inflammatory process

*IRAD* isolated renal artery dissection, *FMD* fibromuscular dysplasia, *SAM* segmental arterial mediolysis, *ANCA* antineutrophil cytoplasmic antibodies, *ANA* antinuclear antibodies, *PET* positron emission tomography

## Conclusion

Isolated renal artery abnormalities, whether vasculitic or dissecting, demand careful diagnostic evaluation with high-resolution imaging and multidisciplinary input.

In both cases, a multidisciplinary approach is crucial, involving nephrologist, rheumatologist, vascular surgeon, and radiologist. Selective renal angiography plays a key role in identifying the dissection flap, which may be not clearly delineated on CT, highlighting the need for stepwise escalation of imaging modalities when diagnostic uncertainty persists.

While vasculitis may require immunosuppression, IRAD often responds well to conservative therapy. The diagnostic overlap and similar clinical presentation of these conditions necessitate vigilance and a tailored approach to management.

## Data Availability

The original contributions presented in the study are included in the article/supplementary material. Further inquiries can be directed to the corresponding author.
